# The Influence of Race and Gender on Staff-Resident Interactions in Nursing Homes

**DOI:** 10.1093/geroni/igaa057.599

**Published:** 2020-12-16

**Authors:** Rachel McPherson, Barbara Resnick, Elizabeth Galik

**Affiliations:** 1 Univesity of Maryland, Baltimore, Maryland, United States; 2 University of Maryland School of Nursing, Baltimore, Maryland, United States; 3 University of Maryland, Baltimore, Maryland, United States

## Abstract

Communication and interactions are an integral part of care in long-term care settings. Resident variables, such as race and gender, shape communication and interaction between staff and residents. The Quality of Interactions Schedule (QuIS) was developed to measure the quality of verbal and nonverbal interactions among nursing staff and older adults initially for those in acute care and later used as well in a variety of long term care settings. A quantified measurement of the quality of interactions between residents and staff was created to quantify the QuIS. The purpose of this study was to describe the gender and racial differences in scored quality of interactions. Data for the present study was based on baseline data from the Evidence Integration Triangle for Behavioral and Psychological Symptoms of Dementia (EIT-4-BPSD) implementation study. A total of 535 residents from 55 settings were included in the analyses. An analysis of covariance was conducted to determine a difference in QuIS scores between males and females while controlling for age. The second model tested for differences in QuIS scores between blacks and whites while controlling for age and gender. There was not a statistically significant difference in QuIS scores between male and female residents. There was a significant difference in QuIS scores between those who were black versus white, such that those who were black received more positive interactions from staff than those who were white. Future work should focus on a deeper examination of resident factors and staff factors that may influence these interactions.

